# Systemic PPARγ Deletion Impairs Circadian Rhythms of Behavior and Metabolism

**DOI:** 10.1371/journal.pone.0038117

**Published:** 2012-08-10

**Authors:** Guangrui Yang, Zhanjun Jia, Toshinori Aoyagi, Donald McClain, Richard M. Mortensen, Tianxin Yang

**Affiliations:** 1 Department of Internal Medicine, University of Utah and Salt Lake Veterans Affairs Medical Center, Salt Lake City, Utah, United States of America; 2 Department of Molecular and Integrative Physiology, University of Michigan, Ann Arbor, Michigan, United States of America; 3 Institute of Hypertension, Sun Yat-sen University School of Medicine, Guangzhou, China; Pennsylvania State University, United States of America

## Abstract

Compelling evidence from both human and animal studies suggests a physiological link between the circadian rhythm and metabolism but the underlying mechanism is still incompletely understood. We examined the role of PPARγ, a key regulator of energy metabolism, in the control of physiological and behavioral rhythms by analyzing two strains of whole-body PPARγ null mouse models. Systemic inactivation of PPARγ was generated constitutively by using Mox2-Cre mice (MoxCre/flox) or inducibly by using the tamoxifen system (EsrCre/flox/TM). Circadian variations in oxygen consumption, CO_2_ production, food and water intake, locomotor activity, and cardiovascular parameters were all remarkably suppressed in MoxCre/flox mice. A similar phenotype was observed in EsrCre/flox/TM mice, accompanied by impaired rhythmicity of the canonical clock genes in adipose tissues and liver but not skeletal muscles or the kidney. PPARγ inactivation in isolated preadipocytes following exposure to tamoxifen led to a similar blockade of the rhythmicity of the clock gene expression. Together, these results support an essential role of PPARγ in the coordinated control of circadian clocks and metabolic pathways.

## Introduction

Most living organisms display behavioral and physiological rhythms in response to the daily changes imposed by rotation of the earth. The rhythms are driven by internal molecular clocks and can be reset by environmental light-dark cycles. The core molecular clock is composed of transcriptional activators and repressors that are assembled into feedback loops [1], [2]. In the simplest form, the heterodimers of transcriptional activators, Bmal1 (brain and muscle aryl-hydrocarbon receptor nuclear translocator-like 1) and CLOCK (the basic helix-loop-helix Per Arnt Sim transcription factors) or its paralog NPAS2 (neuronal PAS domain protein 2), bind to E-box elements of the promoters of target genes and activate gene transcription; the target genes include two families of transcriptional repressors, the Period genes (mPer1–3) and Cryptochrome genes (mCry1 and mCry2), and drive the rhythmic expression [2]–[4]. Upon accumulation in the cytoplasm to a critical level, the proteins of the Per and Cry translocate into the nucleus and repress the transcriptional activity of CLOCK and/or Bmal1, thereby shutting down their own transcription [5]. Additional regulatory loops are interconnected with the positive and negative limbs of the molecular clock providing multiple layers of control of the robustness of oscillation [6], [7]. One such regulatory loop involves the nuclear receptors Rev-erbα and RORα. CLOCK/Bmal1 activate transcription of Rev-erbα, which in turn binds to ROR-responsive element (RORE) in the Bmal1 promoter repressing transcriptional activity of Bmal1 [8]. RORα competes with Rev-erbα to bind the same site, whereas RORα activates Bmal1 transcription [9].

The master regulator of circadian rhythms resides in the suprachiasmatic nucleus (SCN) of the hypothalamus in mammals [10]. The lesion studies published in 1972 demonstrated that electric destruction of the SCN in rats led to a loss of circadian rhythmicity [11], [12]. Subsequent transplantation experiments showed that transplanted SCN restored circadian function in hamster whose own SCN had been ablated [13]. The SCN perceives light and interacts with peripheral clocks through hormonal and neural signals thereby controlling physiological and behavioral rhythms. Various components of the clock system have been identified in peripheral tissues including liver, kidney, heart, and blood vessels [14] and even in immortalized rat fibroblast cells that have been kept in culture for more than 25 years [15]. Approximately 8–10% of the total number of genes expressed in mouse heart and liver exhibit a circadian expression pattern [16]. Moreover, the transcription of only a minority of these circadian genes is driven by systemic hormonal or neuronal signals, whereas the vast majority of them (>90%) are dependent on self-autonomous local circadian oscillators [17], [18].

Growing evidence has emerged to support a physiological link between the circadian rhythms and metabolism. Epidemiological studies showed that perturbations in circadian rhythms in humans involving a shift-working population of 27,485 people are associated with increased risk of obesity and hyperlipidemia [19]. Reduced sleep duration in children is associated with increased risk of being overweight [20]. Studies conducted in mice have also proved the relationship between the circadian rhythms and metabolism. Turek et al. [21] and Rudic et al [22] employing gene knockout mice demonstrate that the disruption of the core molecular clock machinery including Bmal1 and CLOCK leads to hyperphagia and obesity, and metabolic syndrome characterized by hyperleptinemia, hyperlipidemia, hepatic steatosis, and hyperglycemia [21], [22]. At cellular level, Bmal1 is shown to regulate adipose differentiation and lipogenesis in mature adipocytes [23]. Conversely, perturbations of metabolic processes also alter clock function. Kohsaka et al. examined the effect of a high fat diet on behavioral and molecular circadian rhythms in C57BL/6J mice [24]. The high fat fed mice developed impaired circadian rhythms in locomotor activity and metabolism, in parallel with the blunted amplitude of the cyclic expression of clock genes as well as nuclear receptors [24].

Peroxisome proliferator-activated receptor gamma (PPARγ) is a nuclear receptor that heterodimerizes with the retinoid X receptor (RXR) and binds to PPAR responsive elements in the regulatory region of target genes involved in various aspects of metabolism. PPARγ is most abundantly expressed in the adipose tissue where it plays a pivotal role in driving adipocyte differentiation and maintaining adipocyte specific functions, such as lipid storage in the white adipose tissue and energy dissipation in the brown adipose tissue [25]–[30]. In addition, PPARγ is a key regulator of glucose metabolism likely through improvement of insulin sensitivity in metabolic tissues. This insulin sensitizing activity affords the therapeutic potential of PPARγ activation in management of hyperglycemia and insulin resistance in type 2 diabetes. Besides the direct action in the metabolic tissues, PPARγ is recently shown to control lipid metabolism by regulation of microvascular transport of free fatty acids [31]. By analyzing the circadian phenotype of systemic PPARγ null mice, the present study demonstrates a master role played by PPARγ in the control of circadian rhythms in behavior and physiology.

## Results

### The phenotype in constitutive PPARγ KO mice

**Figure 1:**
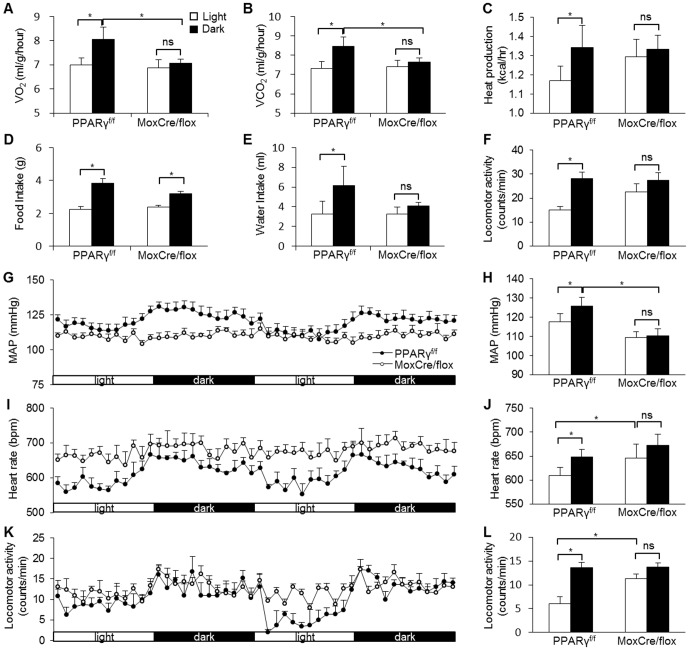
Altered diurnal variation of metabolic and cardiovascular rhythms in MoxCre/flox mice. The canonical diurnal metabolic parameters including VO_2_ (A), VCO_2_ (B), heat production (C), food (D) and water (E) intake, and locomotor activity (F) were measured in MoxCre/flox mice. MAP (G&H), HR (I& J) and locomotor activity (K&L) were recorded using radiotelemetry. N = 5–6 in each group. Data are mean ± SE. *, *p*<0.05; ns, non-significant.

We generated MoxCre/flox mice by crossing floxed PPARγ mice with a transgenic line expressing Cre recombinase under the control of Mox-2 promoter as previously described [30]. The homologous null mice were associated with over 90% lethality at postnatal period and only a small number of them survived to adulthood. VO_2_, VCO_2_, heat production, food and water intake were determined by the four-chamber Oxymax system, and blood pressure (BP) and heart rate (HR) by radiotelemetry; locomotor activity was evaluated by both devices. The four-chamber Oxymax system demonstrated nocturnally activated rhythms in all of the behavioral and metabolic parameters, including VO_2_, VCO_2_, heat production, food and water intake, and locomotor activity in PPARγ^f/f^ mice (Fig. 1A–F). In contrast, the diurnal variations of most of these parameters were nearly absent in MoxCre/flox mice with an exception that the variation of food intake still remained (Fig. 1D). Similarly, by radiotelemetry, PPARγ^f/f^ mice exhibited robust variations of MAP, HR, and locomotor activity, all of which were significantly blunted in MoxCre/flox mice (Fig. 1G–L). Of note, the null mice also developed hypotension, accompanied by tachycardia as previously described (Fig. 1G–J) [30].

### The phenotype in inducible PPARγ KO mice

**Figure 2:**
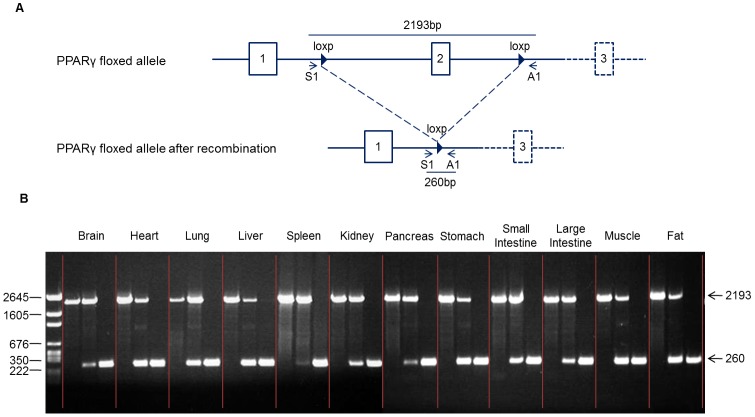
Validation of PPARγ deletion in EsrCre/flox mice. (A) Schematic illustration of primer design for detection of the foxed PPARγ allele (primers S1 and A1). (B) PCR analysis of the floxed PPARγ allele in various tissues of PPARγf/f/TM (left), EsrCre/flox (middle) and EsrCre/flox/TM (right) mice using primers S1 and A1.

In light of the high lethality rate in MoxCre/flox mice, we generated an inducible whole-body PPARγ deletion by using the tamoxifen system. Mating of germ-line floxed PPARγ mice with tamoxifen-inducible Cre-expressing mice produced offspring with inducible homozygous EsrCre/flox mice, which had normal phenotype. To inactivate PPARγ gene, we treated adult EsrCre/flox mice with daily tamoxifen injections for 5 days. We performed PCR analysis of DNA recombination in various tissues from PPARγ^f/f^ and EsrCre/flox mice with or without tamoxifen treatment. We performed PCR analysis of DNA recombination in various tissues from these mice. The DNA recombination was reflected by the loss of the 2193-bp products derived from the floxed allele and appearance of the 260-bp products derived from the recombined allele. The untreated EsrCre/flox mice exhibited partial DNA recombination in most of the tissues possibly reflecting the endogenous steroid activity. After tamoxifen treatment, EsrCre/flox mice had nearly complete DNA recombination in all tissues examined (termed EsrCre/flox/TM) (Fig. 2).

**Figure 3:**
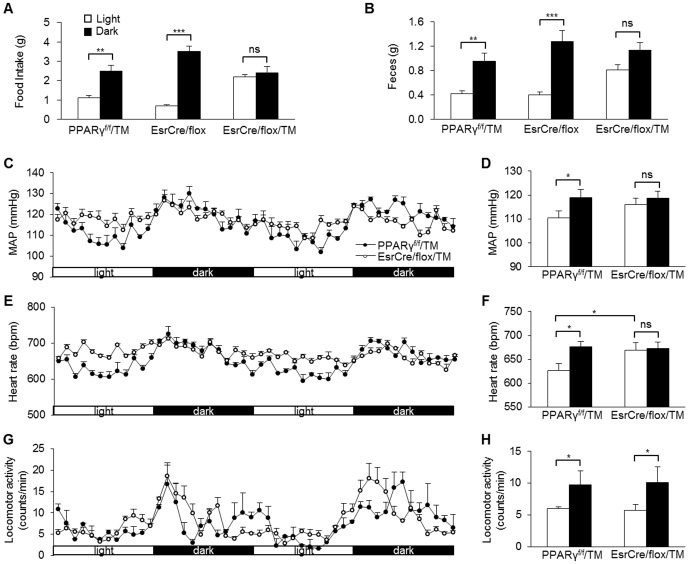
Altered diurnal variation of metabolic and cardiovascular rhythms in EsrCre/flox/TM mice under light/dark cycle. Food (A), and feces (B) were measured during the light and dark phases. MAP (C&D), HR (E&F) and locomotor activity (G&H) were recorded using radiotelemetry for consecutive 2 days. N = 5–6 in each group. Data are mean ± SE. *, *p*<0.05; ns, non-significant.

**Figure 4:**
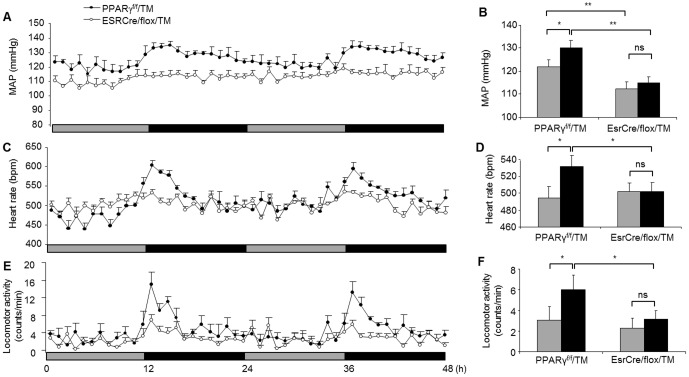
Altered variation of MAP, HR and locomotor activity in EsrCre/flox/TM mice under constant darkness. MAP (A&B), HR (C&D) and locomotor activity (E&F) were recorded using radiotelemetry for consecutive 2 days under constant darkness. Black bars correspond to the period of darkness, and the gray bars indicate the period of subjective light under constant darkness. N = 5–6 in each group. Data are mean ± SE. *, *p*<0.05; **, *p*<0.01; ns, non-significant.

Tamoxifen-treated PPARγ^f/f^ mice (termed PPARγ^f/f^/TM) served as controls. EsrCre/flox/TM mice had normal body weight and were grossly indistinguishable from the floxed controls. Under regular light/dark cycle, PPARγ^f/f^/TM, EsrCre/flox, and EsrCre/flox/TM were placed in metabolic cages (Hatteras Instruments) for measurement of diurnal variations of food and water intake, and feces and urine production. Both PPARγ^f/f^/TM and EsrCre/flox groups displayed obvious day-night variations in food intake and feces production. In contrast, EsrCre/flox/TM mice nearly lost the rhythms of these parameters (Fig. 3A&B). By radiotelemetry, PPARγ^f/f^/TM exhibited rhythms of MAP and HR, both of which were significantly diminished in EsrCre/flox/TM mice as a result of elevated values during the light phase (Fig. 3C–F). However, the variation of locomotor activity was unaffected in the null mice (Fig. 3G–H), suggesting the compensatory mechanisms that might be operated under the light/dark cycle. To address this issue, we examined the influence of constant darkness on the phenotype of EsrCre/flox/TM mice. Under constant darkness, EsrCre/flox/TM mice continued to display the impairment of circadian rhythms of MAP and HR (Fig. 4A–D). It is interesting to note that EsrCre/flox/TM mice developed significant hypotension and bradycardia during constant darkness (Fig. 4A–D). The variation of locomotor activity in these mice during constant darkness was significantly blunted (Fig. 4E&F).

### Evaluation of rhythmicity of clock genes

**Figure 5:**
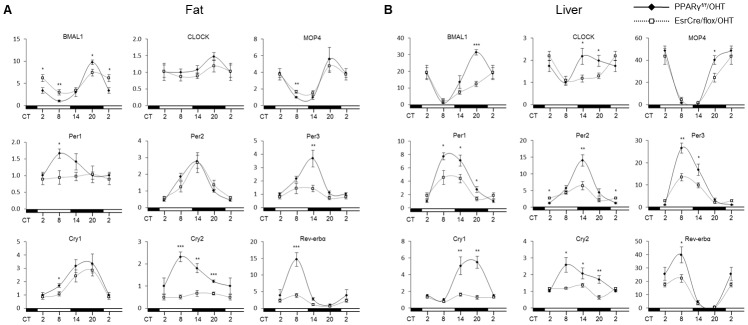
Altered diurnal rhythms of clock gene expression profiles in the fat (A) and liver (B) of EsrCre/flox/TM mice. PPARγ^f/f^/TM and EsrCre/flox/TM mice were sacrificed at 6-h intervals. The epididymal fat and liver were harvested for qRT-PCR analysis of canonical clock gene expression. For each gene, the lowest level of mRNA expression was set to 1. N = 6–8 per group. Data are mean ± SE. *p<0.05, **p<0.01, *** *p*<0.001 versus PPARγ^f/f^/TM mice.

**Figure 6:**
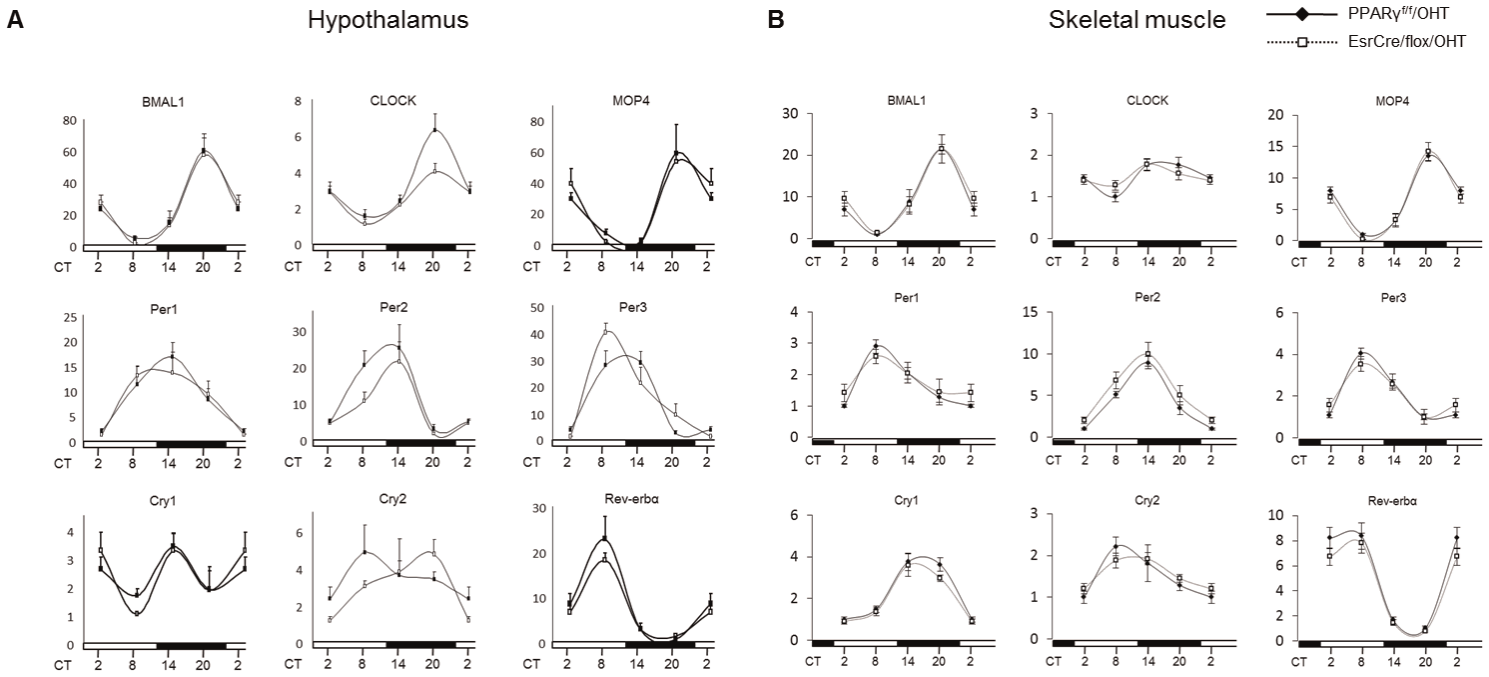
Clock gene expression profiles in the hypothalamus (A) and skeletal muscle (B) of EsrCre/flox/TM mice. PPARγ^f/f^/TM and EsrCre/flox/TM mice were sacrificed at 6-h intervals. The hypothalamus and skeletal muscle were harvested for qRT-PCR analysis of canonical clock gene expression. For each gene, the lowest level of mRNA expression was set to 1. N = 6–8 per group.

We performed qRT-PCR analyses of canonical clock genes in the fat, liver, hypothalamus and skeletal muscle of PPARγ^f/f^/TM and EsrCre/flox/TM mice at various circadian time points under regular light/dark cycles. As expected, adipose expression of canonical clock genes in PPARγ^f/f^/TM mice exhibited robust variations, with Bmal1 and MOP4 peaking at CT20, and Per1, Cry2, and Rev-erbα at CT8, and Per2 and Per3 at CT14 (Fig. 5A) but adipose expression of CLOCK as relatively constant. The expression of most of these clock genes including Bmal1, and MOP4, Per1, Per3, Cry1, Cry2, and Rev-erbα were affected in the fat of EsrCre/flox/TM mice. The changes of most of the clock genes were also seen in the liver (Fig. 5B). Table 1 depicts the amplitudes of canonical clock gene expression in fat and liver of the two genotypes. In contrast, the rhythmicity of the clock genes largely remained intact in the hypothalamus and skeletal muscle of these mice (Fig. 6A&B).

**Table 1:** Peak/Trough ratio of clock gene expression in fat and liver.

	Fat		Liver	
	PPARγ^f/f^/TM	EsrCre/flox/TM	PPARγ^f/f^/TM	EsrCre/flox/TM
Bmal1	8.59±1.22	2.74±0.35***	27.23±4.33	7.89±1.77***
CLOCK	1.47±0.12	1.37±0.21^ns^	2.17±0.36	2.30±0.22^ns^
MOP4	5.671±1.453	3.191±0.5312^ns^	48.75±3.96	26.95±4.49**
Per1	1.66±0.14	1.16±0.28^ns^	7.72±0.53	2.48±0.69***
Per2	6.49±0.91	4.57±1.01^ns^	14.03±1.69	2.76±0.40***
Per3	2.84±0.66	2.00±0.54^ns^	26.58±2.26	4.08±0.77***
Cry1	3.36±0.71	3.96±0.58^ns^	5.48±0.71	1.99±0.37***
Cry2	2.32±0.22	1.37±0.27*	2.58±0.44	2.12±0.18^ns^
Rev-erbá	19.89±2.80	6.58±1.11**	39.88±5.99	49.59±9.82^ns^

Shown are mean ± SE.

* *p*<0.05,

** *p*<0.01,

*** *p*<0.001 versus PPARγf/f/TM.

ns: no significant difference.

### PPARγ regulation of clock gene expression in preadipocytes and the role of 15-deoxy-Δ12,14-prostaglandin J_2_

**Figure 7:**
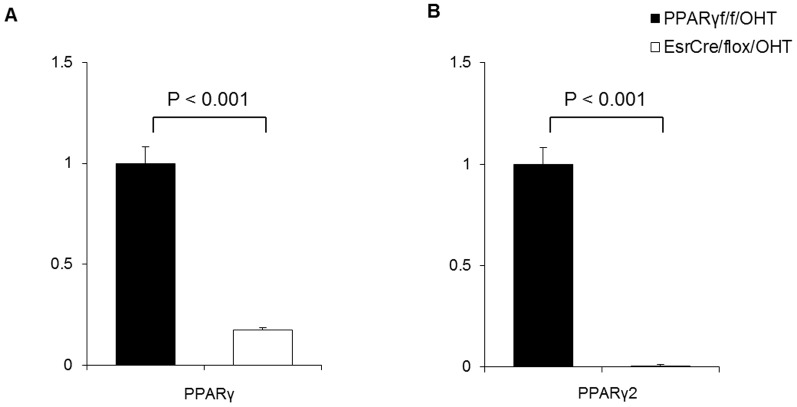
Validation of PPARγ deletion in 4-OHT-treated preadipocytes. (A) qRT-PCR analysis of total PPARγ expression in preadipocytes. (B) qRT-PCR analysis of PPARγ2 expression in preadipocytes.

**Figure 8:**
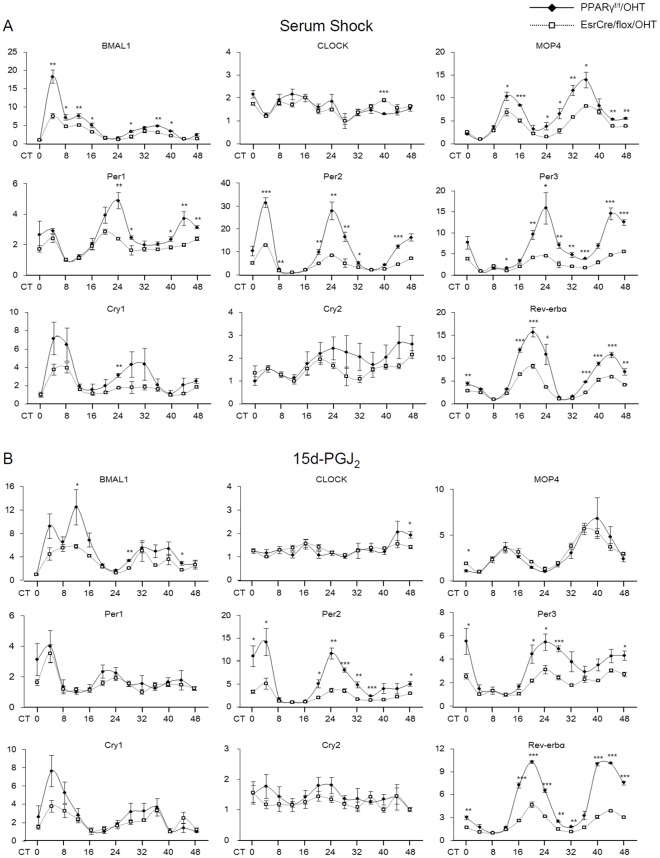
Serum shock or 15d-PGJ_2_-induced clock gene expression profiles in PPARγ-deficient preadipocytes. 4-OHT-treated primary preadipocytes from PPARγ^f/f^ and EsrCre/flox mice (termed PPARγ^f/f^/OHT and EsrCre/flox/OHT, respectively) were stimulated with 50% horse serum (A) or 10 µM 15d-PGJ_2_ (B) for 2 h. Cells were harvested at 4-h intervals for 48 h and subjected to RNA extraction and qRT-PCR analysis of the canonical clock gene expressions. For each gene, the lowest level of mRNA expression was set to 1. N = 4 in each group/time point. Shown are mean ± SE. *p<0.05, **p<0.01, *** *p*<0.001 versus wild-type control.

To investigate whether PPARγ directly regulated the clock system, we used the tamoxifen system to produce PPARγ deletion in primary preadipocytes and examined the consequence in expression of the clock genes. Exposure of EsrCre/flox preadipocytes to 4-hydroxytamoxifen (4-OHT) for 2 days resulted in 83% decrease of total PPARγ mRNA level (Fig. 7A) and nearly complete deletion of PPARγ2 (Fig. 7B) as assessed by qRT-PCR. 4-OHT-treated PPARγ^f/f^ preadipocytes served as controls (PPARγ^f/f^/OHT). In the control cells, 50% horse serum treatment triggered the rhythmic expression of most of clock genes for 48 h. In contrast, in vitro PPARγ inactivation led to a significant blockade of the rhythmic expression of Bmal1, MOP4, Per1–3 and Rev-erbα, and, to the less extent, in the rhythmic expression of CLOCK, Cry1 and Cry2 (Fig. 8A).

15-deoxy-Δ12,14-prostaglandin J_2_ (15d-PGJ_2_), a natural ligand of PPARγ, has been reported as an entrainment factor for the circadian clocks [32]. Next, we examined the expression profiles of clock genes in the primary culture of preadipocytes stimulated by 15d-PGJ_2_. 15d-PGJ_2_ triggered the rhythmic expression of clock genes in the control preadipocytes, to an extent almost comparable to 50% horse serum (Fig. 8B). In contrast, the rhythmic expression of Bmal1, Per2, Per3 and Rev-erbα genes was significantly reduced in PPARγ-deficient cells (Fig. 8B).

**Figure 9:**
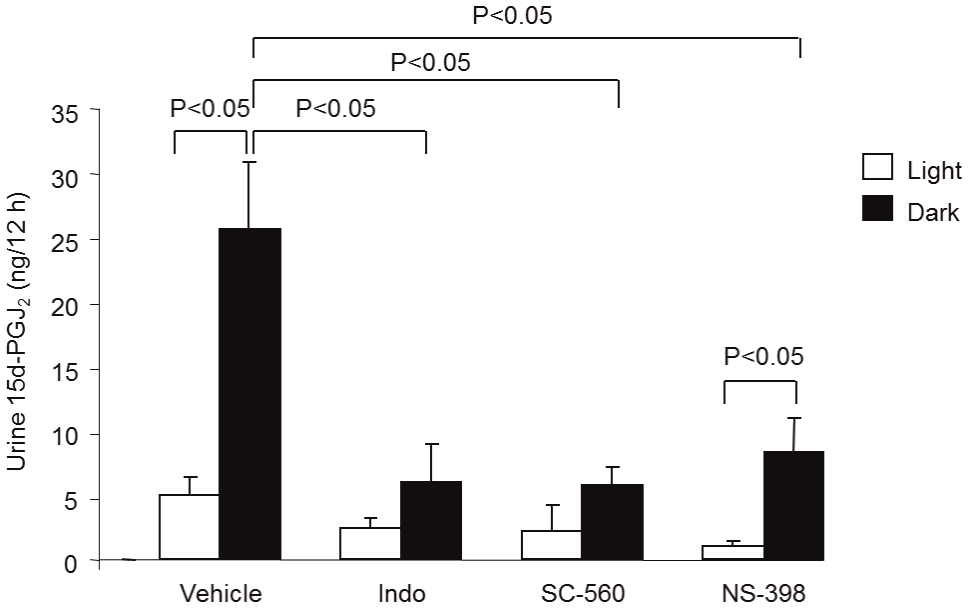
12-h urine output of 15d-PGJ_2_ during the light and dark phase in PPARγf/f mice treated with DMSO (vehicle), indomethacin (Indo) (5 mg/kg/d), SC-560 (30 mg/kg/d), or NS-398 (5 mg/kg/d) for 3 days. The compounds were administered from diet and dosing was based on estimated food intake. N = 4–7 in each group. Data are mean ± SE.

We employed ELISA to determine urinary excretion of 15d-PGJ_2_. The specificity of 15d-PGJ_2_ ELISA has been validated by testing cross activities with other prostanoids; the values were: 0.11% for PGD_2_, 0.1% for delta12-PGJ_2_, 0.05% for PGA_2_, and 0% for TXA_2_, PGI_2_, and PGE_2_. Urinary 15d-PGJ_2_ excretion was found to exhibit robust diurnal variation that was effectively attenuated by indomethacin and the COX-2 inhibitor SC-560; the COX-2 inhibitor NS-398 reduced the baseline level of urinary 15d-PGJ_2_ but failed to influence the magnitude of the diurnal variation (Fig. 9).

## Discussion

A large body of evidence from human and animal studies has demonstrated that the regulation of molecular clocks is linked to pathways of energy metabolism. A better understanding of the molecular basis of the relationship between the molecular clocks and metabolism may shed light on the etiologies as well as therapies of metabolic diseases. PPARγ is a key regulator of energy metabolism and is best known for serving as a therapeutic target for management of type 2 diabetes. Despite the intensive investigation, the mechanism of how PPARγ achieves an integrative control of energy metabolism is not fully understood. We hypothesize that PPARγ may function as an integrator of the molecular clocks and metabolism. Since this function may involve the multi-faceted interaction of PPARγ in multiple tissues, the use of generalized knockout models is necessary. The germline knockout of PPARγ produces the embryonic lethality due to abnormal placenta vascularization, hepatic dysfunction and multiple hemorrhages [33], [34]. The embryonic lethality was rescued by breeding Mox2-Cre mice with floxed PPARγ mice so that PPARγ deletion was restricted to the embryo but not trophoblasts [30]. Unfortunately, these null mice exhibited a high incidence of postnatal death (∼90%) possibly as a result of developmental abnormalities. To circumvent this issue, we created a mouse model of inducible PPARγ deficiency by using the tamoxifen system. Non-tamoxifen-treated PPARγ^f/f^ Esr1-Cre mice had normal growth and morphology indistinguishable from floxed controls although they exhibited partial DNA recombination in various tissues. In contrast, upon tamoxifen treatment, these mice had nearly complete DNA recombination in all tissues examined. In this way, the embryonic or postnatal lethality seen in the prior models was completely prevented. The availability of the inducible PPARγ null model offers a powerful tool for investigating the physiological function of PPARγ in adulthood.

The most novel finding of the present study was the robust alteration of circadian rhythms in a spectrum of physiological, metabolic and behavioral parameters of the two strains of systemic PPARγ null mice. Under regular light/dark cycles, MoxCre/flox mice displayed a nearly complete loss of circadian rhythms of food and water intake, metabolism (VO_2_, VCO_2_, and heat production), cardiovascular parameters (BP and HR) and locomotor activity. The variations of most of these parameters in EsrCre/flox/TM mice were blunted under both light/dark or constant darkness conditions with an exception for the locomotor activity. The rhythm of the locomotor activity in these mice remained intact under light/dark cycle but was diminished under constant darkness. The reason for the difference in the rhythm of the locomotor activity between the genotypes is unclear but one confounding factor may come from the high lethality rate in young MoxCre/flox mice. Despite this limitation, the circadian phenotypes of the two strains of PPARγ null models generated by different methods are largely consistent, establishing an essential role of PPARγ in the control of rhythmicity of behavior and physiology. Emerging evidence has demonstrated a physiological link between the circadian rhythms and metabolism [35], [36]. Our results strongly suggest that such a link is at least in part mediated by PPARγ. Of note, besides the change in the circadian rhythm, EsrCre/flox/TM mice exhibited reduced MAP and HR when switched from regular light/dark cycle to constant darkness, suggesting an additional role of PPARγ in light-dependent regulation of cardiovascular function. A possibility exists that PPARγ activation may help sustain sympathetic activity especially in the absence of light.

The robust circadian phenotype of the two strains of whole-body PPARγ null mice suggests a non-redundant role of this nuclear receptor in the circadian regulation. Indeed, emerging evidence supports a direct coupling of PPARγ with Bmal1. Our previous study demonstrates that PPARγ directly regulates Bmal1 transcription in the vascular cells, thereby regulating the cardiovascular rhythms [37]. Here, we found that systemic inactivation of PPARγ led to blunted rhythmicity of Bmal1, along with Per and Cry genes, in adipose tissues and liver but not skeletal muscle. These disparate roles were consistent with the report that PPARγ expression was found rhythmically expressed in mouse adipose tissue and liver but not skeletal muscle [38]. This phenomenon was further recapitulated by in vitro inactivation of PPARγ in cultured preadipocytes. Interestingly, the rhythmicity of these clock genes in the knockout hypothalamus remained intact. These findings support a direct interaction between PPARγ and the canonical clock system in the peripheral tissues, but not in the hypothalamus.

Our results also suggest that besides direct transcriptional regulation of Bmal1, PPARγ may determine the robustness of Bmal1 oscillation via Rev-erbα, a negative regulator of Bmal1 [36]. In parallel with the changes in canonical clock gene expression, the oscillation of Rev-erbα expression in both adipose tissues and liver was remarkably suppressed in PPARγ null mice in vivo as well as in PPARγ-deficient preadipocytes in vitro. These findings agree with the observation that Rev-erbα expression cycles in adipose tissue [39] and induced during adipogenic process following PPARγ activation by rosiglitazone [40], [41].

The study of Nakahata et al. employed an unbiased approach, namely the in vitro real-time oscillation monitoring system to identify unknown entrainment factors for clock genes in cultured 3T3 cells (Nakahata et al. 2006). Among 299 peptides and bioactive lipids tested in this study, 15d-PGJ_2_ was identified as a novel entrainment factor that produces the most robust effects on rhythmicity. In agreement with this observation, we found that a single treatment with 15d-PGJ_2_ produced robust rhythmicity. However, a difference between the two studies exists concerning the involvement of PPARγ. The present study demonstrated that tamoxifen-induced PPARγ deletion remarkably blunted the rhythmicity in preadipocytes exposed to 15d-PGJ_2_. This finding argues against the Nakahata's study reporting independence of the 15d-PGJ_2_ action from PPARγ based on the use of the PPARγ antagonist DW9662. Of note, the similar blockade of clock gene expression was observed in PPARγ-deficient preadipocytes exposed to 50% horse serum and 15d-PGJ_2_ with a few exceptions. For example, the blockade of Cry1 and MOP4 by PPARγ deletion was observed after serum shock but not after 15d-PGJ_2_. These results suggest a different mechanism responsible for regulation of Cry1 and MOP4 under the current experimental condition.

15d-PGJ_2_ was initially identified as an endogenous PPARγ ligand based on data from several *in vitro* systems [42], [43]. Subsequently, this notion was challenged by the observation that the production of 15d-PGJ_2_ in several mammalian tissues, as measured by mass-spectroscopy, was several orders of magnitude below the levels required for in vitro activation of PPARγ [44]. However, increasing evidence suggests that 15d-PGJ_2_ covalently binds to multiple proteins including NF-kappaB, AP1, p57, thioredoxin, as well as its receptor PPARγ [45]–[48], raising a possibility that 15d-PGJ_2_ may mainly exist in the bound rather than free form. This may explain the detection difficulty with mass- spectrometry. Using enzyme immunoassay, we found that the production of 15d-PGJ_2_ was subjected to circadian regulation. The effective inhibition of the diurnal variations with indomethacin and SC-560 but not NS-398 strongly suggests that COX-1 activity is responsible for generating 15d-PGJ_2_. In line with this observation, COX-1 deficiency leads to an attenuation of the circadian variations in BP, HR, and sympathetic activity [49].

In summary, the two strains of whole-body PPARγ null mice consistently develop blunted physiological and behavioral rhythms. The impaired rhythmicity of the canonical clock genes in the null mice was found in adipose tissues and liver but not skeletal muscles or the kidney. PPARγ inactivation in isolated preadipocytes resulted in a similar blockade of the rhythmicity. Together, our studies have defined PPARγ as a key integrator of molecular clocks and metabolism.

## Materials and Methods

### Transgenic mouse lines

PPARγ^f/f^ mice contain two loxP sites inserted into intron 1 and 2 of the PPARγ gene flanking the critical exon 2 (Akiyama et al. 2002). The floxed mice were crossed with MoxCre mice [30], [50] and Cre/Esr mice (Jackson Laboratories, Bar Harbor, ME), respectively, to yield mice heterozygous for both floxed PPARγ and Cre. The second crossing of heterozygous mice to PPARγ^f/f^ mice yielded homozygous floxed PPARγ mice with heterozygous Cre gene (termed MoxCre/flox and EsrCre/flox). Genotypes were confirmed by PCR analysis as described previously [37]. For in vivo experiments, 4 to 6-month old male mice were maintained under 12:12 hr L/D cycle. All procedures were in accordance with the guidelines approved by the University of Utah Institutional Animal Care and Use Committee.

### Tamoxifen administration

Tamoxifen stock solution was prepared as previously described [51] with modifications. Briefly, 100 mg tamoxifen (Sigma) was suspended in 150 µl of ethanol followed by the addition of 850 µl of corn oil (Sigma). This 100 mg/ml tamoxifen solution was aliquoted and stored at −20°C. The solution was thawed at 55°C before use. PPARγ^f/f^ mice and EsrCre/flox mice were administered with 50 µl (5 mg) of tamoxifen solution per day by oral gavage for 5 consecutive days (termed PPARγ^f/f^/TM and EsrCre/flox/TM, respectively). All experiments were conducted at least 10 days after the last tamoxifen administration.

### Evaluation of DNA recombination of PPARγ

DNA recombination of the PPARγ gene was evaluated in the brain, heart, lung, liver, pancreas, stomach, intestine, spleen, kidney, muscle and fat from PPARγ^f/f^/TM, EsrCre/flox and EsrCre/flox/TM mice. Primers flanking the 2 loxP sites and exon 2 were used to amplify a product of 2193 bp from the floxed allele and 260 bp from the recombined allele.

### Metabolic studies

Regular metabolic cages (Hatteras Instruments, Cary, NC) were used for urine and feces collections and also for measurement of food and water intake during the light and dark phases. Indirect calorimetry was performed with a four-chamber Oxymax system (Columbus Instruments, Columbus, OH). Animals were allowed to adapt to the metabolic chamber for 4 h and then food and water intake, movement, oxygen consumption (VO_2_), carbon dioxide output (VCO_2_) and heat production were measured every 15 min for 3 days from individually housed mice.

### Telemetry recordings

Under general anesthesia, the radiotelemetric device (model No. TA11PA-C20, DSI, MN) was implanted through catheterization of the carotid artery as previously described [52]. Following 1-week recovery, the device was turned on for continuous recording of blood pressure, heart rate and locomotor activity for 48 h. The recording was made during regular light/dark cycle or constant darkness.

### Analysis of circadian gene expression

PPARγ^f/f^/TM and EsrCre/flox/TM mice were killed at 6 hr intervals of 24 hr. The fat, liver, skeletal muscle and kidney were harvested for qRT-PCR analysis of canonical clock genes including Bmal1, CLOCK, MOP4, Cry1–2, Per1–3 and Rev-erbα. The primer sequences are listed in supplemental table 1. qRT-PCR amplification was performed using the SYBR Green Master Mix (Applied Biosystems) and the Prism 7500 Real-Time PCR Detection System (Applied Biosystems). The oligo sequences are shown in Table 2. Cycling conditions were 95°C for 10 min followed by 40 repeats of 95°C for 15 s and 60°C for 1 min.

**Table 2:** Primers for qRT-PCR.

Gene name	Sense Primer (5′-3′)	Antisense Primer (5′-3′)	Accession No.
Bmal-1	GGAAATACGGGTGAAATCTATG	TTCTGCGAGGTGTCCTATGT	NM_007489
CLOCK	TTGCGTCTGTGGGTGTTG	TGCTTTGTCCTTGTCATCTTCT	NM_007715
Cry1	CTGATGTATTTCCCAGGCTTT	GCTGTCCGCCATTGAGTT	NM_007771
Cry2	ATGTGTTCCCAAGGCTGTTC	GGTTTCTGCCCATTCAGTTC	NM_009963
Per1	TCCTCAACCGCTTCAGAGAT	TGGGAGACATAGCAGGGAGT	NM_011065
Per2	GTTTGCTGTGGCTGTGTCC	TCTCATTCTCGTGGTGTTTCC	NM_011066
Per3	ATGTGGGCCAACAGCTCTAC	GGGAGGCTGTAGCTTGTCAG	NM_011067
MOP4	TCCCTGGTAACACTCGGAAA	GCCATCTAATGCCTCCAACA	NM_008719
Rev-erbα	CTTCCGTGACCTTTCTCAGC	CAGCTCCTCCTCGGTAAGTG	NM_145434
PPARγ	TTTTCAAGGGTGCCAGTTTC	AATCCTTGGCCCTCTGAGAT	NM_001127330
PPARγ2	CTCCTGTTGACCCAGAGCAT	CAACCATTGGGTCAGCTCTT	NM_011146
GAPDH	GTCTTCACTACCATGGAGAAGG	TCATGGATGACCTTGGCCAG	M32599

### Primary culture of preadipocytes

White adipose tissues from 3-week old PPARγ^f/f^ and EsrCre/flox mice were used for preadipocyte culture. The epididymal and inguinal fat depots were dissected, minced, and transferred to a Krebs-Ringer buffer (Sigma, K4002) containing 15 mM sodium bicarbonate, 10 mM HEPES, 2 mM sodium pyruvate and 1% BSA (pH 7.4). Collagenase type I (Worthington Biochemical) was added at 2 mg/ml. The tissues were shaken at 100 rpm, 37°C for 60 min. The digest was filtered through a 70-µm nylon filter (BD Falcon). The flow-through was centrifuged (100× g, 5 min) and the cell pellet was suspended in the DMEM. The cells were recentrifuged, resuspended in regular medium, 5% fetal bovine serum (FBS)-DMEM and cultured in flasks. Culture medium was exchanged after 24 hours and every 2 days thereafter. Before experiments, cells were seeded into 24-well plates at 1×10^5^/well. After confluence, cells were treated with 100 nM 4-hydroxytamoxifen (4-OHT, Sigma) for 2 days followed by medium exchange with fresh regular medium. Then the cells were challenged with serum shock or 15d-PGJ_2_. Briefly, at time = 0, the medium was exchanged with DMEM supplemented with 50% horse serum or 10 µM 15d-PGJ_2_, and after 2 hr, this medium was replaced with regular medium. At the indicated times, the cells were harvested in TRI Reagent (Applied Biosystems) and applied for RNA extraction. These RNA samples were used for qRT-PCR analysis of circadian genes.

### 15d-PGJ_2_ assay

PPARγ^f/f^ mice treated with DMSO (vehicle), indomethacin (Indo) (5 mg/kg/d), SC-560 (30 mg/kg/d), or NS-398 (5 mg/kg/d). The compounds were administered from diet and dosing was based on estimated food intake. After treatment for 3 days, urine was collected during the light phase (ZT0–12) and dark phase (ZT12–24) and was stored at −80°C before the assays. Urinary 15d-PGJ_2_ were measured by using a commercial EIA kit (Assay Designs, Ann Arbor, MI).

### Statistical Analysis

All values are presented as mean ± SE. ANOVA and Bonferroni post-tests were used for comparisons among multiple groups and the unpaired Student's t test for comparisons between two groups. Differences were considered to be significant when the *P* value was less than 0.05.
